# The Implications of EFL/ESL Teachers' Emotions in Their Professional Identity Development

**DOI:** 10.3389/fpsyg.2021.755592

**Published:** 2021-09-13

**Authors:** Ling Cheng

**Affiliations:** ^1^School of Humanities and International Education and Exchange, Anhui University of Chinese Medicine, Hefei, China; ^2^School of Liberal Arts, Shinawatra University, Bangkok, Thailand

**Keywords:** teachers' professional identity, teachers' emotions, teachers' beliefs, teacher development, teacher education program

## Abstract

The leading implementers of the curriculum and educational system are teachers, so the success and failure of the educational system depend mainly on its teachers. If teachers have an established professional identity, it leads to the success of the educational system. Professional identity, like other aspects of the teaching and learning process, is influenced by various factors. Investigating this concept requires identifying the factors affecting it. One of the most important factors that influence teachers' professional identity is teachers' emotions. Teachers' emotions also can have a significant impact on teachers' performance. After searching the databases, this review article examines the role of teachers' emotions and their professional identities in English as a foreign language (EFL) or English as a second language (ESL) classrooms. This review paper unpacks that factors such as teachers' pedagogical beliefs, their positive and negative emotional experiences, their environmental and cultural factors, and their perceptions and expectations of these conditions could affect their emotions as well as their professional identity. Teachers' identity is shaped through ongoing negotiation and interaction that encompasses their personal and professional lives. Taking these factors into account in teacher training courses might notify teachers of the challenges that they might have in their classrooms and provide them with practical solutions.

## Introduction

Due to the growing globalization and the simultaneous spread of English throughout the world, the study of socio-cultural and political factors of education in general and English Language Teaching (ELT) in particular has become one of the necessities of today's international societies (Kanno and Stuart, 2011; De Costa and Norton, 2017). The purpose of education in today's communities is not only to transfer knowledge from the old generation to the new generation and to strengthen the mental powers of learners but also to develop a comprehensive understanding of effective communication in meaningful ways (Liu and Xu, 2011; Norton and De Costa, 2018). The development of a society and its comprehensive development depend on the educational system of that society and the teachers of that system (Tsui, 2007; Lee et al., 2013). A teacher is one of the key stakeholders of this sensitive profession in any educational system (Derakhshan et al., 2020a). Improving teacher quality is a key element in improving education (Timoštšuk and Ugaste, 2010). However, research shows that teachers especially EFL/ESL teachers during the first few years of their professional life quit their jobs at high rates. One of the reasons for this could be the lack of a desirable professional identity in the EFL/ESL field of study (Lee and Jo, 2016).

Such development requires interested, compassionate, efficient, professionally qualified, and well-formed EFL/ESL teachers (Karlsson, 2013). Educational policymakers believe that identity is one of the basic pillars of achieving the macro goals of the educational system (Pillen et al., 2013; Henry, 2016). Thus, investigating teachers' professional identity helps us to understand who teachers are and how they operate, and how to move through the various social, cultural, political and economic discourses that have permeated their workplace (Beauchamp and Thomas, 2009). To investigate an EFL/ESL teacher's professional identity, we must first know what identity is. The teachers' professional identity has been investigated by many researchers since 1970 (Henry, 2016). The origin of the identity of EFL/ESL teachers depends on their education in teacher education programs (Sutherland and Markauskaite, 2012). The interest in investigating identity arises from epistemological and methodological changes from cognitive and psychological approaches of those that design critical and social frameworks of teacher education programs (Stenberg, 2010).

## Review of Literature

Professional identity, like other aspects in the teaching and learning process, is influenced by a variety of factors inside and outside EFL/ESL teachers, among which their emotions cannot be overlooked. The database searching shows the role of teacher's emotions and their professional identities in EFL/ESL teaching practices.

### Teachers' Professional Identity

The concept of identity has different definitions in the literature. Besides, the concept of professional identity is used in various ways in teacher education programs (Lutovac, 2020; Wang and Derakhshan, 2021b). Marcia (2002) defines professional identity as follows: Professional identity is the measurement of personal skills and values related to active employment and exploration, followed by job commitment. Based on this criterion, Marcia divides professional identity into four categories: (1) Successful identity, (2) Early identity, (3) Late identity and (4) Confused identity. Successful identity includes people who are committed to professional goals and values through interaction with the environment and involvement in professional exploration. Early identity refers to those individuals who have committed to professional values and goals through their adaptation to the beliefs and attitudes of others, such as parents and other important people (Lee et al., 2013; Pillen et al., 2013). Late identity refers to the identity of those who seek professional interests, values and perspectives in order to achieve professional commitment. Finally, the confused identity describes individuals who are committed to professional values and goals by engaging in professional exploration. Marcia's quadruple identity is not necessarily a different and exclusive stage of a growing chain. It is not fixed in nature, but it may change over time. Marcia (2002) refers to the skills and abilities as well as the professional interests of individuals as important indicators in the formation of their professional identity. He points out that if this identity is created, the person will be able to establish a deep and mutual relationship with the environment and society and have a sense of unity and connection about himself and his profession, and also to achieve inner and psychological satisfaction in his teaching environment.

Some studies have linked the concept of professional identity with teachers' concepts or perceptions of themselves (Beauchamp and Thomas, 2009; Chong and Low, 2009; Dang, 2013). These studies have shown that teachers' attitudes about themselves are strongly influenced in determining how they teach, how they progress as teachers and their attitudes toward educational change (Akkerman and Meijer, 2011; Lee et al., 2013; Lutovac, 2020). The formation of professional identity is a topic that is mentioned in many sources as one of the vital points for the success of any language context (Lee and Yin, 2010; Kanno and Stuart, 2011). It is often seen as a struggle because EFL/ESL teachers need to understand different and sometimes competitive perspectives, expectations and roles they must face or adapt to (Cheng et al., 2009; Derakhshan et al., 2020a).

Previous studies have demonstrated the essential role of teaching practice and reflective activities in shaping teachers' identities (Liu and Xu, 2011; Sutherland and Markauskaite, 2012; Norton and De Costa, 2018). Emphasis on professional identity in the early stages of the career can help educators to overcome the multidimensionality and complexity of the teaching profession so that they can contribute to their and students' success in the language environments (Chen, 2019a). If identity is built into the discourses and contexts in which teachers work, teachers' identities can be determined in advance (Tsui, 2007; Xie and Derakhshan, 2021). However, it is important to note that identity formation is not a fixed action and does not occur at certain times (Lutovac, 2020). Teachers create their identity through a process of ongoing negotiation, debate, and dialogue that includes both their personal and professional lives (Tsui, 2007).

Therefore, professional identity is an ongoing process in which new experiences and the interpretation of these experiences play a significant role. In general, it is assumed that the development and formation of teacher identity never stops and can be considered a lifelong process (Chong and Low, 2009). In addition, professional identity is influenced by both the person and the context (Dang, 2013). This means that the professional identity of the teacher is not completely unique. Professional teachers are expected to think and act professionally, but no doubt not only their professional characteristics but also their personal and environmental characteristics are involved in their decision making (Pillen et al., 2013; Chang-Kredl and Kingsley, 2014). In general, it is stated that professional identity is dynamic. However, how teachers deal with these characteristics varies depending on the value they place on them personally (Stenberg, 2010). Dang (2013) explains further and believes that teachers' professional identities are clearly not individualized and unchangeable. Language teachers may try to maintain their habits and routines, but they are subconsciously influenced by external factors (Henry, 2016).

From another point of view, the teacher's professional identity includes sub-identities that are more or less harmonious (Chang-Kredl and Kingsley, 2014). Teachers' sub-identities originate from their different contexts and relationships in the language teaching environment. Some of these sub-identities may be broadly linked and can be considered as the core of teachers' professional identities (Pishghadam et al., 2019; Wang and Derakhshan, 2021b). However, some of them might be in consistent with other identities, and some of them might be in complete conflict with them. It seems essential for a teacher that these sub-identities be balanced and not in conflict with each other. During primary teacher training, teachers often experience such conflicts.

Although many studies have been conducted on teachers' professional identities, it is not yet possible to determine how and what specific characteristics shape teachers' identities (Lutovac, 2020). Chen (2019a) considered identity as an experience in relation to the three modes of belonging that are interaction, imagination, and balance. There are many theories about how identity is formed, for example, Identity Discourse Theory (IDT) that emphasizes identity is formed mainly through language (De Costa and Norton, 2017). Another view considers identity construction through participation in meaningful social activities (Fathi and Derakhshan, 2019). The formation and growth of professional identity are the processes of maturity of an individual that begin before and during the learning of a profession (for instance as an EFL/ESL teacher) and continue to grow until the individual is recognized as an expert in that profession (Lee and Yin, 2010; Kanno and Stuart, 2011).

One of the factors affecting the teachers' professional identity is individuals' events and past experiences. Experiences from childhood, education, dramatic life events, etc. are effective in developing the EFL/ESL teachers' professional identity. These factors include aspects of life that are outside of the professional context (Chang-Kredl and Kingsley, 2014). In other words, teachers' personal and family life experiences that might include their school experiences and interactions during this period can be considered as one of the effective factors in developing their professional identity.

Another factor that might play a significant role in shaping teachers' professional identity is the teacher education program (Lutovac, 2020). Teacher training programs have an important impact on the formation and development of teachers' professional character. University environment and its leadership style, teachers' teaching experiences, job duties and workplace characteristics, and attachment to the job and the emotional environment of the workplace are among the subcategories of this important factor that are also effective in developing teachers' professional identity. In other words, physical, emotional, and social dimensions of teachers' workplace can motivate or demotivate teacher students (Sutherland and Markauskaite, 2012).

Numerous studies have revealed that in all these processes, from childhood to school experience, from individual life to teacher education programs, and from teacher education programs to the actual teaching environment or classroom, teachers' emotions play a central role in shaping teachers' professional identity (Cross and Hong, 2012; Chen, 2016; Barcelos and Aragão, 2018; Dewaele and MacIntyre, 2019).

### Teachers' Emotions

Recently, many studies have been conducted on why paying attention to emotional factors can support the language teaching/learning process (Cowie, 2011; Bahia et al., 2013; Barcelos and Aragão, 2018; Wang and Derakhshan, 2021a; Wang et al., 2021). Dewell and Paulsco (2019) point out that emotions play an astonishing role in English language teaching and learning. This can cause more concerns for EFL/ESL learning environments and the internal interactions of EFL/ESL teachers and learners. It is not always possible to provide a clear definition of this factor. But when dealing with emotions in the second language or foreign language learning process, what we are dealing with is primarily about the emotional, mood, or attitudinal aspects that affect teachers' and learners' behavior (Taxer and Frenzel, 2015; Miri and Pishghadam, 2021). Yoo and Carter (2017) focused on a language class and the impact of teachers' emotions on different aspects of a language class. They believe that success in learning a second or foreign language depends less on the materials, techniques, and language analysis, and more on what goes on inside and between people (teacher-students and students-students relationships) in the classroom.

Inside each teacher includes individual factors such as attitudes, teaching motivations, communication motivations, self-esteem, anxiety, boredom, and teaching methods that greatly affect the way they teach and interact in language classes (Aragao, 2011; Derakhshan et al., 2021; Pishghadam et al., 2021). In addition to the learner's talent, these factors can affect the development of language skills. These relationships exist not only between students and between teacher-students, but also between teachers and students and the target language and culture (Becker et al., 2014). The importance of emotions in learning a second language and a foreign language cannot be denied (Chen, 2016). Emotions have both positive and negative impacts (Yoo and Carter, 2017). The positive impact of emotions can lead to more effective learning, and the negative impact can close the mind and prevent students from learning (Chen, 2019b). So we need to look for ways to both avoid negative reactions and create a positive emotional atmosphere in the classroom among our students.

There is a relationship between learning and emotional factors in any situation in the classroom, but it is very important in language learning because if students cannot control the language completely, they can be less self-confident and therefore not learn the subject matter well (Cross and Hong, 2012). This can affect learning in many ways. If there are disorders related to learners' identities, learning becomes more complex, so teachers must consider the emotional aspect of language learners and avoid possible obstacles in the cognitive process (Farouk, 2012). For the past two decades, researchers have paid special attention to the teachers' emotions as one of the influential areas in English language classes (Cross and Hong, 2012; Yoo and Carter, 2017; Chen, 2019a). Teachers' professional identity development research has focused more on examining rational factors such as teacher knowledge, teacher skills, and capacities (Golombek and Doran, 2014). Although teachers' emotions are just as important as other factors, the impact of this factor on the development and formation of teachers' identities and students' learning has generally been overlooked.

According to researchers, emotions are a mysterious human phenomenon that has remained an unsolved mystery to researchers for many years (Lanas and Kelchtermans, 2015; Fathi et al., 2021; Greenier et al., 2021). Horwitz (2001) was the first to speculate that non-native language teachers are prone to Foreign Language Anxiety (FLA). She believes that anxiety in these teachers arises from the irrational analysis of their abilities in the target language. Contrary to what many studies have suggested, anxious teachers do not always have a foreign language deficit and are less fluent in the target language than their less anxious counterparts. She believes that anxiety is more prevalent among perfectionist teachers in language learning that tend to recognize and magnify small and insignificant shortcomings in the target language production. Horwitz (2001) believes that teachers who pursue an ideal and unrealistic level of expertise in the target language are more likely to be concerned about their competence as second-language or foreign-language teachers. Chen (2019a) stated that emotions as a social construct have been built in the society in which the teacher lives and teaches. Teachers' professional identity stems from conscious or subconscious judgments about their success in achieving goals or maintaining standards or beliefs during interactions.

Research on teachers' emotions has gained more attention in recent years (Nichols et al., 2017). The studies highlighted that teachers' emotions affect all teachers' actions such as teachers' behavior, teaching methods, professional identity, personal life, their educational changes, and students' behavior and learning (Timoštšuk and Ugaste, 2012). There are numerous studies conducted on teachers' emotions, most of which were qualitative studies that used semi-structured interviews as their data collection instruments (Bloomfield, 2010; Anttila et al., 2016; Zhu, 2017; Derakhshan et al., 2020b). A quantitative study can also be valuable to the relevant studies. In a more comprehensive view, Farouk (2012) stated that teachers' emotions consist of three levels. The first level is the dynamic mental state of teachers, the second level is the ability of emotional self-regulation, and the third level is their responses to external and internal stimuli.

Teachers' emotions are internal and hidden emotions that are expressed in communication and interaction with students, their parents, and their colleagues. This means that the environment is an integral element of teachers' emotions (Chang-Kredl and Kingsley, 2014). Thus, it can be said that teachers' emotions are directly related to the environment; it means that teachers' emotions do not exist independently in a particular teacher or environment, but are expressed through interaction between the person and the environment (Benesch, 2012; Derakhshan et al., 2019). Understanding and instilling these emotions provides an extraordinary opportunity for teachers to make a real difference in the lives of their students, but it is important to note that these changes also affect the lives and even the performance of the teachers (Thomas and Beauchamp, 2011; Chen, 2019b). Teachers' emotions have great potential for both strengthening interpersonal relationships in EFL/ESL classrooms and developing extracurricular activities outside of the classrooms. This feeling can even create opportunities for students to learn and teach in different situations (Anttila et al., 2016). Lack of positive or negative emotions can limit teacher education processes (Zhu, 2017).

### Teacher Professional Identity and Teachers' Emotions

Although researchers have proposed several definitions as well as different measurement criteria for assessing teacher identity (e.g., Beijaard et al., 2004), they all agree on two salient features of teacher identity. The first characteristic is that identity is not examined but is a process that is constantly interpreted (Sutherland et al., 2010), it means that a person is not evaluated, but his reaction to an emotional act is analyzed. The second prominent feature is the emergence of identity in the interaction of teachers with students and understanding of the environment in which they work.

Many studies have focused on the professional identity development program (Bijard et al., 2004; Benesch, 2012; Timoštšuk and Ugaste, 2012). They have identified four different groups of emotions among foreign language teachers: (1) energy, excitement, and passion; (2) internal conflict, frustration and discouragement; (3) vulnerability, interaction and hope; and (4) generosity, gratitude and inspiration (Zhu, 2017).

The results of these studies show that any change, correction or improvement in education is associated with different emotions. Regarding the identity of novice teachers, Anttila et al. (2016) proved that teaching is not just a technical task and teachers should also be familiar with psychological issues. This acquaintance includes emotional experiences that provide outstanding information about how teachers' identities evolve. Importantly, these studies have proven that there is an interrelationship between emotional experiences and professional identity. Not only the identities of novice teachers affect their actions and emotions, but their actions and emotions also affect the formation of their professional identity (Thomas and Beauchamp, 2011).

## Conclusion

Reviewing the literature, the researchers have found that studies that focus on teachers' emotions in EFL/ESL classrooms clearly show positive results for teaching. Recent studies clearly show that good cognitive performance and effective learning are strongly influenced by emotional factors. To achieve successful teaching in the classroom, it is very important that language teachers pay special attention to the emotional factors and teacher students and student-student relationships. A review of these studies showed that the relationship between teacher identity and emotion is not one-way or linear. Rather, they are inextricably linked to each other through a continuous, multidirectional interactive process. For example, when teachers experience certain unpleasant emotions, these emotions may challenge and alter the existing identities related to their beliefs about teaching. This performance is not just about unpleasant emotional experiences. Pleasant emotional experiences may also give rise to emerging identities (Cross and Hong, 2009). Similarly, incoming emotions can affect how subsequent emotional reactions occur or are interpreted (Yoo and Carter, 2017). Therefore, new methods should be sought for both avoiding negative reactions and creating positive feelings in the classroom among our students.

### Implications for EFL/ESL Classrooms and Teacher Education Programs

One important way to pay more attention to the relationship between teachers' emotions and their professional identity is to create new activities in EFL/ESL class. Although there is a lot of content in textbooks, sometimes teachers can generate new activities and investigate their impact on students' emotions. Among the goals that are considered in doing these exercises are to increase students' motivation, improve their self-esteem, help them to achieve a better understanding of each other, and the ability to do group works.

These types of activities can also be very positive for teachers' progress because if teachers work with only one textbook, teaching will not be so creative and interesting for them and their students. Thus, teachers can become more autonomous and sometimes creative and generate activities to improve their professional identity and students' learning (Sutherland et al., 2010). In this way, they can have a closer relationship with their students and their experiences. Cross and Hong (2012) emphasizes that teachers can use humanitarian activities to complete, review, and introduce their existing content. They do not mean abandoning the syllabus but suggest that by adding meaningful and personal activities, they can increase students' interaction and interest and cultivate their positive emotions. In general, teachers should follow their own curriculum, but they can go beyond the textbook and do any exercise that increases productivity and strengthens teaching. Using the knowledge of their students, teachers can create activities that are appropriate and interesting to their students. By understanding their emotions, teachers can also help promote their students' independence and motivation.

In some studies language classes were taught with a focus on the emotional factors, using neither a textbook nor a pre-determined curriculum. In the end, the language skills of the students were clearly better than the control groups, which were taught in conventional ways (Nichols et al., 2017). The students in the experimental groups had significant motivation for the class and also showed significant improvements in factors such as self-confidence, creativity, willingness to speak in front of others, ability to control their own learning, and autonomy. Therefore, positive relationships with students along with paying attention to the development of language skills can create effective and coherent classes.

One of the questions that every teacher may have in teaching is how much they should involve their emotions in the process of managing and controlling the classroom. Two important points should be considered about a teacher's emotions. The first is to moderate and control them. The learning environment is like a chemistry lab for students. They need to learn how to behave in the classroom, without witnessing teachers' explosions. In other words, teachers should expect any behavior from their students and be prepared to face different situations without reaching the blast point. Second, teachers should base their emotions on students' success and achievements, not on students' emotions in the classroom or their mood. Students' emotions are always fluctuating, and teachers need to control their emotions in the face of these fluctuations. The relationship between learning and emotional factors should be balanced, because, in language learning, if students cannot control the language completely, they might be less confident with themselves and therefore not have a better understanding of the subject matter (Cross and Hong, 2012). It is the same with teachers' emotions toward students' learning. In other words, teachers should not react violently and unpredictably to students' behavior and always treat them with care. It is suggestive that teachers encourage students more, and be more patient and positive in the classroom.

An emotionally stable teacher is someone who can gain students' trust by controlling and communicating with them. Only in the long run can the desired result be achieved if a balance is struck between the emotions of teachers and their students. The success of in EFL/ESL classroom depends more on what goes on between teacher and students (Yoo and Carter, 2017). Behavior cannot be controlled in any situation with fluctuating emotions. Teachers' emotions have been studied from different aspects. Various studies have shown that teachers generally have three categories of characteristics: First, mastery of specialized knowledge of the subject of teaching. Second, mastery of the components of a teacher's job, including teaching with all its components, from lesson plans to active teaching methods and proper assessment methods. However, the third category of characteristics is the character of a teacher or the personal characteristics of a teacher.

It is generally believed that any expert teacher, even if he or she has good skills in teaching methods, will not be an effective teacher unless they add the characteristics of the third type to their teaching. In this view, the influential element that communicates is the teachers' view of their works, which can be very effective in the effectiveness of their activities. The most influential teachers were not preferably teachers who had a lot left in their students' minds from their level of literacy, but teachers who paid a lot of attention to them as human beings, for teachers' identity is always created through a process of ongoing negotiation, debate, and dialogue including their personal and professional lives as well (Tsui, 2007). They have been with their students and the students have felt this companionship.

From the findings of the current study, teacher educators can discover the importance of teachers' emotions in shaping teachers' identities in EFL/ESL classrooms. The study showed that teachers' positive or negative emotional experiences that conflict with novice teachers' beliefs have a significant impact on identity formation. There are some critical points that should be considered in designing any effective teacher education program that promotes teachers' professional identity: (1) teachers' pedagogical beliefs, (2) teachers' positive and negative emotional experiences, (3) the teaching environment and culture, and finally (4) teachers' understanding and judgment about themselves and these three vital points. Environmental and cultural factors and their relationship with teachers' professional identity can be highlighted in future studies.

## Author Contributions

The author confirms being the sole contributor of this work and has approved it for publication.

## Funding

This study was sponsored by the pedagogical projects both funded by Anhui Provincial Educational Department, China (Grant Nos. 2020jyxm1028 and 2020jyxm1034) and the key pedagogical project funded by Anhui University of Chinese Medicine (Grant No. 2021zlgc040). This study was also sponsored by TCM Language Service Base Project of Anhui Province, China (Grant No. 2020AH0020).

## Conflict of Interest

The author declares that the research was conducted in the absence of any commercial or financial relationships that could be construed as a potential conflict of interest.

## Publisher's Note

All claims expressed in this article are solely those of the authors and do not necessarily represent those of their affiliated organizations, or those of the publisher, the editors and the reviewers. Any product that may be evaluated in this article, or claim that may be made by its manufacturer, is not guaranteed or endorsed by the publisher.
